# Influence of the Size of the Field of View on Visual Perception While Running in a Treadmill-Mediated Virtual Environment

**DOI:** 10.3389/fpsyg.2019.02344

**Published:** 2019-10-16

**Authors:** Martina Caramenti, Paolo Pretto, Claudio L. Lafortuna, Jean-Pierre Bresciani, Amandine Dubois

**Affiliations:** ^1^Department of Neurosciences and Movement Sciences, University of Fribourg, Fribourg, Switzerland; ^2^Istituto di Bioimmagini e Fisiologia Molecolare, Consiglio Nazionale delle Ricerche, Segrate, Italy; ^3^HumanTech Institute, University of Applied Sciences and Arts Western Switzerland, Fribourg, Switzerland; ^4^Virtual Vehicle Research Center, Graz, Austria; ^5^Istituto di Fisiologia Clinica, Consiglio Nazionale delle Ricerche, Milan, Italy; ^6^LPNC, University Grenoble Alpes, Grenoble, France; ^7^Université de Lorraine, 2LPN-CEMA Group (Cognition-EMotion-Action), EA 7489, Metz, France

**Keywords:** virtual reality, visual speed perception, treadmill running, field of view, optical flow, locomotion

## Abstract

We investigated how the size of the horizontal field of view (FoV) affects visual speed perception with individuals running on a treadmill. Twelve moderately trained to trained participants ran on a treadmill at two different speeds (8 and 12 km/h) in front of a moving virtual scene. Different masks were used to manipulate the visible visual field, masking either the central or the peripheral area of the virtual scene or showing the full visual field. We asked participants to match the visual speed of the scene to their actual running speed. For each trial, participants indicated whether the scene was moving faster or slower than they were running. Visual speed was adjusted according to the responses using a staircase method until the Point of Subjective Equality was reached, that is until visual and running speed were perceived as matching. For both speeds and all FoV conditions, participants underestimated visual speed relative to the actual running speed. However, this underestimation was significant only when the peripheral FoV was masked. These results confirm that the size of the FoV should absolutely be taken into account for the design of treadmill-mediated virtual environments (VEs).

## Introduction

Because of the associated physiological and psychological benefits, physical activity (PA) is fundamental for human health and well-being. Physical activity notably helps to reduce the risk of many diseases and improving the functional status and the quality of life. Treadmills constitute one of the most widely used pieces of equipment to train cardiovascular fitness indoors. However, treadmills have some shortcomings. Specifically, treadmill locomotion is monotonous and can easily lead to boredom. Moreover, it fails to reproduce overground locomotion because it is characterized by a sensory discrepancy between kinesthetic/motor and visual information (Pelah and Barlow, 1996). Coupling treadmills and virtual environments (VEs) could contribute to improve the engagement and effort of the user, and to enhance the physical experience. In addition, it would help minimizing the kinesthetic-visual discrepancy.

Though coupling fitness equipment with VEs likely represents the future of cardiovascular training equipment, some issues must still be resolved. This is notably the case regarding motion perception. In particular, when moving around an environment, perceived body motion results from the integration of motion cues provided by different sensory channels. Visual and proprioceptive cues, together with efference copy information, are among the most prominent sensory inputs in this integration process. Regarding more specifically treadmill-mediated VEs, though one might legitimately assume that visual speed should match locomotion speed, i.e., the speed at which the belt is moving, studies have repeatedly shown that walkers/runners tend to underestimate optical flow speed relative to locomotion speed (Thurrell et al., 1998; Banton et al., 2005; Durgin et al., 2005a; Thurrell and Pelah, 2005; Kassler et al., 2010; Powell et al., 2011; Caramenti et al., 2018). In particular, these studies have shown that visual speed needs to be set higher than the actual walking/running speed to be perceived as matching.

For devices that should reach a wide number of users, such as treadmills, an additional related problem regards the size of the display, and more particularly the horizontal field of view (FoV). Specifically, several studies have shown that the size of the FoV affects visual speed perception, the underestimation of which tends to increase as the FoV decreases (Van Veen et al., 1998; Nilsson et al., 2014, 2017). These studies were performed for different motion-related activities, such as walking, cycling, or simulated driving. For instance, Van Veen et al. (1998) found the visual perception of cycling speed to depend on the peripheral FoV. In this study, visual speed was underestimated when the FoV was smaller than 73 degrees, and slightly overestimated when the FoV was larger than 107 degrees. With individuals walking at 3 mph (4,83 km/h) and gazing straight ahead, Banton et al. (2005) found a 50% underestimation of walking speed with a FoV of 50 degrees. In the same study, walking speed was accurately perceived when peripheral flow was maximized with downward or sideways gaze (Banton et al., 2005). With a 90-degree FoV, Thurrell et al. (1998), Thurrell and Pelah (2002) found that a 3 mph (4,83 km/h) walking speed is perceived about 20% slower than it actually is, i.e., it is underestimated by 20%. Note that when the FoV reduces, visual speed underestimation tends to increase. In particular, with a FoV of about 90 degrees, visual gains (i.e., visual speed/locomotor speed) that are perceived as natural range from 1.67 to 2.03, whereas with a FoV of 25 degrees, the visual gains that are perceived as natural rather range from 2.14 to 2.64 (Nilsson et al., 2014). Taken together, these results suggest that while walking, visual speed is perceived more accurately as peripheral flow increases. Similar results were observed with static individuals. In particular, in an experiment in which perceived visual speed was investigated with seating individuals, Pretto et al. (2009) found that at FoVs smaller than 60 degrees, visual speed is underestimated with a bias that is inversely related to the size of the visible area. Interestingly, these authors also found that visual speed tends to be overestimated if the optical flow is presented in peripheral vision only. Note that in this study, the horizontal FoV was 220 degrees and optical flow was also provided on the floor (quarter sphere screen).

Surprisingly, to date, no study has investigated how the size of the FoV affects visual speed perception with running participants. Yet, in order to design treadmill-mediated visual environments for a widespread diffusion, it is fundamental to understand how the display dimensions could affect perceived speed, notably so that the user can experience the best possible perceptual coherence.

Here we used a virtual environment and psychophysics methods to assess how the size of the FoV affects the perceived speed of the optical flow with participants running on a treadmill.

## Materials and Methods

Twelve moderately trained to trained subjects (1 female, 11 male) with a median age of 25.53 (26.04 ± 2.37 SD) participated in the study. All were naïve about the purpose of the study, had normal or corrected-to-normal vision and none had a history of cardiovascular disease. All participants gave their informed and written consent prior to the inclusion in the study, which was performed in accordance with the ethical standards specified by the 1964 Declaration of Helsinki. This study was approved by the Ethics Committee of the University of Fribourg.

Before performing the running task, the participants filled out the short version of the International Physical Activity Questionnaire (IPAQ – French version), which estimates the weekly amount of PA expressed in MET-min/week. METs, or metabolic equivalents, are defined as the ratio between the energy rate expended during a specific activity and the energy expenditure at rest (1MET). In other words, METs describe the energy expenditure of an activity. The volume of PA per week is calculated as the sum of the METs expended in different activities over a week, taking into account the time in minutes spent in each activity. This volume is expressed as MET-min/week.

**FIGURE 1:**
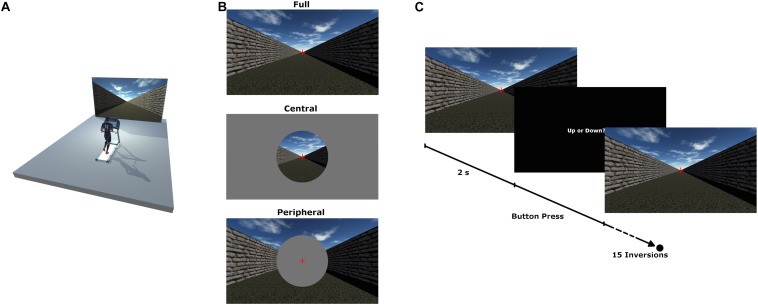
Experimental setup **(A)**, experimental conditions **(B)** and experimental design **(C)**.

Participants ran on a treadmill (HP Cosmos Mercury - Running surface: 150 cm by 50 cm) located in front of a 430 cm × 270 cm projection screen. A 2D virtual scene representing an open-air hallway was projected onto the screen (Projector: Barco F50 WUXGA, Resolution: 1920 × 1200 pixel) to simulate optical flow (see Figure 1A). The virtual scene was created using Unity 3D (Unity Technologies). When running, the participants’ head was located 300 cm from the center of the screen, with an effective FoV of 70° in full screen mode. The room was darkened during the experiment, with the display screen being the only source of light. While running, participants held a custom-made plastic cylinder (115 mm × 30 mm, 15g) in each hand, with a response button located on the top surface. This allowed participants to send their responses via Bluetooth to the computer by effortlessly pressing on the buttons with their thumb while running.

The experiment consisted of six blocks. In each block, the participants ran at one of two different speeds (i.e., treadmill speed), namely 8 km/h or 12 km/h. For each treadmill speed, three different FoV conditions were presented. Specifically, different masks were implemented in the visual scene to manipulate the visible area shown on the screen. Note that a fixation cross positioned at the end of the hallway was visible for the whole duration of the trials. The fixation cross was located at the focus of expansion of the optical flow resulting from the relative motion between the visual scene and the observer (i.e., participants). In the full FoV condition, the open-air hallway was visible on the whole screen. In the central FoV condition, the hallway was displayed only within a circular mask of 30°centered on the fixation cross. Finally, in the peripheral FoV condition, a disk covered the central portion of the screen (30°) so that optical flow was visible only in the outer area of the screen, i.e., in peripheral vision (see Figure 1B). The order of presentation of the six blocks was counterbalanced between subjects using a Latin square.

For each FoV condition, the participants were presented with the visual scene while they were running at constant speed on the treadmill. For each trial, the task was to estimate if the visual scene was slower or faster than the actual running speed. Specifically, participants were instructed to gaze at the fixation cross that was visible at the end of the virtual hallway. The visual scene was presented for 2 s before the participants were asked the question “Up or down?” displayed on a black screen:

• “Up” if they perceived visual speed to be slower than the treadmill, requiring an increase of the speed of the virtual scene - left response button.
• “Down” if they perceived visual speed to be faster than the treadmill, requiring a decrease of the speed of the virtual scene – right response button.

Participants gave their response by pressing the corresponding switch while continuing to run on the treadmill. Once the participant pressed the response button, the graphics returned from the black screen to the visual scene of the following trial (see Figure 1C). Luminance values were 6.7 cd/m^2^ for the visual scene used as the main stimulus and 0.2 cd/m^2^ for the black screen. For each trial, the speed of the visual scene was adjusted with a one up one down staircase method (Leek, 2001; Kingdom and Prins, 2010) that took into account the previous responses of the participant. The staircase defined an increase/decrease of the visual speed of 0.5 Km/h until the first inversion of the response, followed by steps of 0.3 Km/h. When 15 inversions of the responses were reached the staircase session was stopped. This method was used to determine the Point of Subjective Equality (PSE), namely a perceptual threshold indicating the respective values of two quantities when they are perceived as being equivalent. For each running speed, the PSE indicated the speed of the visual scene (optical flow) that was perceived as matching the actual running speed. For each of the two running speeds, two consecutive staircase sessions were proposed in a random order, the staircase session starting once with a higher (+4 km/h) and once with a lower (−4 km/h) visual speed than the treadmill speed. At the end of each staircase session, the participants could take a short pause before starting the next staircase session.

Prior to the experiment, the participants had a few minutes to familiarize themselves with treadmill running at different running speeds. Using a training program, they were then familiarized with the experimental setting and task. The training program presented the visual scene at different speeds. Experimental trials were initiated when the participant felt comfortable running at different speeds.

## Results

**FIGURE 2:**
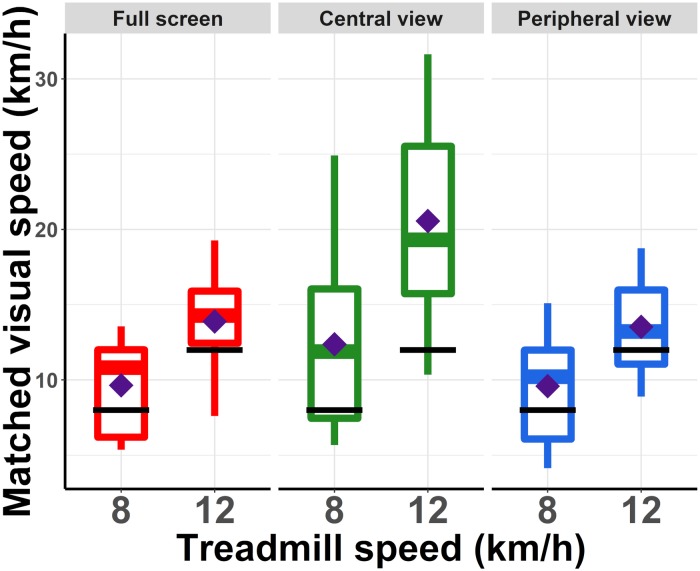
Speed of the visual scene that was perceived as matching treadmill speed. In all conditions for most participants and on average (filled diamonds), the visual scene had to move faster than the actual treadmill speed (black lines) to be perceived as matched.

For each FoV condition (i.e., Full screen, Peripheral view, Central view) and each treadmill speed (i.e., 8 and 12 km/h), perceived visual speed was compared to the actual running speed using either a one-sample *t*-test or a Wilcoxon signed–rank test (when perceived visual speed data was not normally distributed). Please refer to Supplementary Table S1 for the data. After assessing normality using a Shapiro–Wilk test, the one-sample *t*-test was used when perceived visual speed data was normally distributed, while the Wilcoxon signed–rank test was used for not-normally distributed data. For all six tests, the alpha level was corrected for multiple comparisons using Holm correction. For all six conditions, the visual speed was set higher than the actual running speed, meaning that the optical flow had to move faster than the treadmill speed for the two speeds to be perceived as matching, indicating that visual speed was underestimated relative to running speed. However, this underestimation was significant only in the Central view condition at a running speed of 12 km/h (*t*(11) = 4.323, *p* = 0.001). It also barely failed to reach significance in the Central view condition at a running speed of 8 km/h (*t*(11) = 2.666, *p* = 0.02 uncorrected, with an alpha adjusted to 0.01). For the other four conditions, there was no significant difference between the set visual speed and the actual treadmill speed. Figure 2 shows the average matching visual speed for the three FoV conditions and the two treadmill speeds.

**FIGURE 3:**
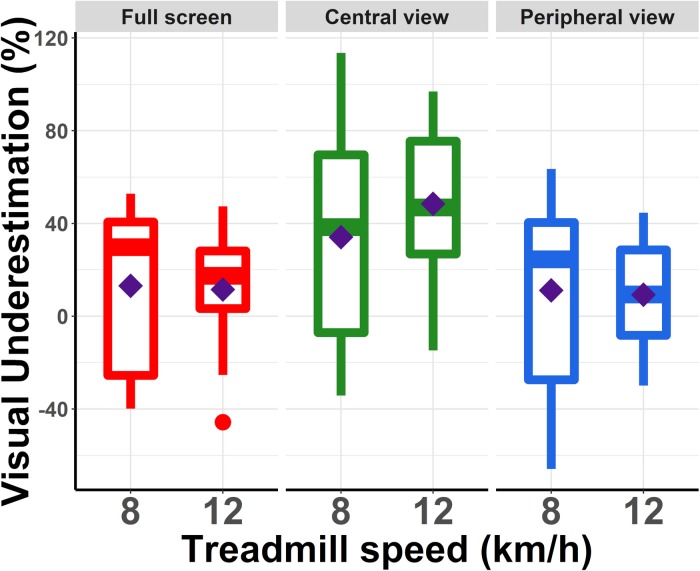
Percentage of underestimation of visual speed relative to running speed. These values were computed using the equation: *ln (perceived visual speed/actual running speed) ^∗^ 100*. Each box summarized for each running speed and each condition the distribution of the responses of the participants. The diamond corresponds to the mean value. The central line corresponds to the median, with the box defining the inter-quartile range (IQR) between the first and the third quartile and the whiskers corresponding to ± 1.5IQR.

We then tested whether there were significant differences across conditions regarding the amplitude of relative underestimation of visual speed (see Figure 3). For each FoV condition and each treadmill speed, we computed the percentage of visual under/overestimation using the equation: *ln (perceived visual speed/actual treadmill speed)^∗^100*. This percentage indicates how much slower (negative values) or faster (positive values) the optical flow had to move relative to the actual running speed, i.e., treadmill speed, for the two speeds to be perceived as equivalent. The logarithm (i.e., *ln*) was used so that neither visual nor treadmill speed was used as an absolute reference value (Graff, 2014).

The mean relative underestimation values measured in the different conditions were compared using a linear mixed model. We used a linear mixed model because we had a repeated measures design and data was non-parametric. The analysis revealed a main effect of the FoV (χ^2^(2) = 33.50, *p* < 0.0001). In particular, visual speed underestimation was larger for the Central view condition (41.30%) than for the Full screen (12.32%) and Peripheral view condition (10.22%). There was no main effect of the treadmill speed (χ^2^(1) = 1.02, *p* = 0.31), and visual underestimation varied from 19.45% at 8 km/h to 23.11% at 12 km/h.

Planned orthogonal contrasts indicated that the visual underestimation observed in the Central view condition was of significantly larger amplitude than those observed in the Full screen (*p* = 0.003) and Peripheral view condition (*p* = 0.0014), respectively. On the other hand, the amplitude of visual underestimation was not significantly different between the Full screen and the Peripheral view condition (*p* = 0.76).

**FIGURE 4:**
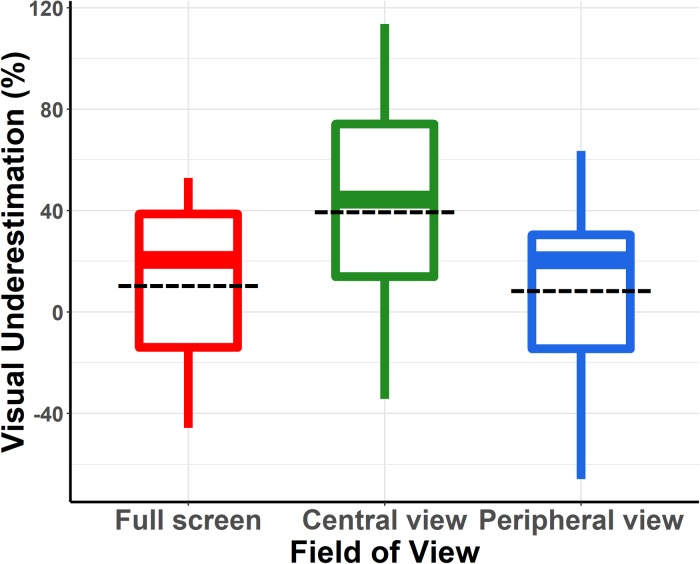
Percentage of underestimation of visual speed relative to running speed in the three FoV conditions (when collapsing the two running speeds). These values were computed using the equation: *ln (perceived visual speed/actual running speed) ^∗^ 100*. Each box summarized for each running speed and each condition the distribution of the responses of the participants. The dashed line corresponds to the mean value. The central line corresponds to the median, with the box defining the inter-quartile range (IQR) between the first and the third quartile and the whiskers corresponding to ± 1.5IQR.

Because the linear mixed model analysis revealed no significant effect of treadmill speed, we collapsed the two treadmill speed levels and compared the three FoV conditions using a Friedman Rank Sum test (repeated measures and non-parametric data). The analysis indicated a main effect of the FoV on visual speed underestimation (χ^2^(2) = 15.167, *p* < 0.001), thereby confirming the outcome of the linear mixed model analysis. *Post hoc* tests confirmed a significant difference between the Central view and the other two conditions, whereas the Full screen and Peripheral view conditions did not differ from one another (see Figure 4).

## Discussion

While running on a treadmill in front of a moving virtual scene, participants were asked to match the visual speed of the scene to their running speed (i.e., treadmill speed) in different FoV conditions. Previous studies have shown that the size of the FoV affects speed perception, but this had never been tested with running subjects. The results of the current study show that for all three FoV conditions, namely Full screen, Peripheral view and Central view, and both running speeds tested in the experiment (i.e., 8 and 12 km/h), visual speed tended to be underestimated with respect to running speed. Specifically, the speed of the virtual scene was systematically set higher than the actual running speed in order to be perceived as matching treadmill speed. This relative underestimation of visual speed was significant only in the Central view condition at a running speed of 12 km/h, while it barely failed to reach significance after correction for multiple comparisons in the Central view condition at a running speed of 8 km/h.

The visual underestimation (relative to running speed) we observed in the Central View condition is significantly larger than the underestimation observed in the Full screen and in the Peripheral view condition. This is in line with the results reported in previous studies performed with cycling (Van Veen et al., 1998) and walking individuals (Thurrell et al., 1998; Thurrell and Pelah, 2002; Banton et al., 2005), but also with sitting-still participants (Pretto et al., 2009). When moving forward, the angular velocity of the optical flow is relatively low in the part of the visual field directly surrounding the focus of expansion. In most situations, and definitely in our experiment, this area is the central part of the visual field. In contrast, the angular velocity of the optical flow is much higher in peripheral vision because this usually corresponds to an area that is farther to the focus of expansion. In line with this, the underestimation of visual speed that we observed in the Central view condition likely results from the fact that in this condition, only the low angular velocity component of the optical flow was visible. This confirms that the extent of the FoV should absolutely be considered when designing VEs.

Several studies have suggested that peripheral vision is important for motion perception, affecting speed perception (Pretto et al., 2009, 2012), navigation abilities (Czerwinski et al., 2002; Turano et al., 2005), and the sensation of self-motion deriving from a moving stimulus (Brandt et al., 1973; Berthoz et al., 1975; Mohler et al., 2005). Peripheral vision has also been shown to play an important role in evoking vection (i.e., the subjective experience of self-motion without actual self-motion) (Brandt et al., 1973; Berthoz et al., 1975). In their 2009 study, Pretto et al. (2009) found that when the central part of the visual field was masked (in the range 10 to 60 degrees, which is the range also tested in our experiment), the speed of the presented optical flow was systematically overestimated. In contrast to their observations, we did not find any overestimation of visual speed in the Peripheral view condition, and this condition did not differ from the Full screen condition. This difference between their results and ours could be linked to the dimensions of the screen (i.e., FoV) used in the two experiments. In particular, Pretto et al. (2009) used a quarter sphere screen with a 230° horizontal × 125° vertical FoV, with projection on the floor. This is much larger than the projection screens used in most studies as well as than the maximum FoV of head mounted displays. The results of Van Veen (Van Veen et al., 1998) seem to confirm this hypothesis, since this author also found a slight overestimation of the speed of the optical flow when the FoV was larger than 107°.

An additional difference between our study and that of Pretto et al. (2009) is the motion-related sensory information that was available during the simulated body motion. In our experiment, participants were running on a treadmill. Therefore, in addition to the optical flow, participants were provided with motion-related proprioceptive and efference copy information. The task of the participants was actually to compare visual information with proprioceptive and efference copy information. As opposed to this, in the study of Pretto et al. (2009), participants were sitting still. In other words, in their study, optical flow was the only source of motion information. This difference between the two studies might explain, at least partially, why Pretto and colleagues observed an overestimation of visual speed in the Peripheral FoV condition, whereas we did not. Specifically, in our experiment, proprioceptive and efferent copy information might have prevented participants from overestimating visual speed by indicating them that they were not running that fast. Several studies have shown that visual, proprioceptive and vestibular information is used to update the representation of the visual space for an efficient navigation, providing congruent information about the displacement (Warren and Hannon, 1988; Loomis et al., 1996, 1999; Chance et al., 1998; Yardley and Higgins, 1998; Sun et al., 2004a; Turano et al., 2005; Jürgens and Becker, 2006; Souman et al., 2009). In fact, studies have shown that when either one of visual, vestibular or proprioceptive information is missing during locomotion, it can be predicted based on the other two sensory channels (Mittelstaedt and Mittelstaedt, 2001; Durgin and Gigone, 2007; Durgin, 2009). For the perception of visual speed, evidence suggests that optical flow speeds near to walking speeds are better discriminated when one is walking (Durgin et al., 2004; Durgin, 2009), but it is not clear if vision or proprioception is more important for monitoring self-motion speed when both are available (Sun et al., 2004b). This means that the manipulation of visual, vestibular or proprioceptive information can influence the perception of self-motion and the related motor activity (Durgin et al., 2005b; Mohler et al., 2007; Pelah et al., 2015).

A last difference between our study and that of Pretto et al. (2009) relates to the experimental design. Specifically, Pretto and colleagues used a Two-interval forced choice (2 IFC) in which the Full screen condition constituted the reference and was used as standard stimulus. In other words, perceived visual speed in the other FoV conditions was always measured relative to perceived visual speed in the Full screen condition. In contrast to that, in our experiment, perceived visual speed was compared to the actual running speed. In theory, this methodological difference should not have a strong impact on the difference between the Full screen and the other FoV conditions. However, and in line with the above-mentioned arguments, speed comparison was visuo-visual in Pretto et al. (2009), whereas it was visuo-proprioceptive + efference copy in our experiment. This might have affected the way optical flow information was processed.

Previous studies with walking (Banton et al., 2005; Durgin et al., 2005a; Kassler et al., 2010; Powell et al., 2011) and running participants (Caramenti et al., 2018, 2019) reported a general tendency to underestimate visual speed when compared to locomotor speed. Our results confirm the tendency to underestimate visual speed, as the mean visual-locomotor gain observed in our study ranged from 1.31:1 at 8 km/h to 1.34:1 at 12 km/h. One factor that could have affected the visual-locomotor gain is treadmill running in itself, which has been shown to influence speed perception. In fact, treadmill locomotion is usually perceived as faster than the corresponding overground speed (White et al., 1998; Marsh et al., 2006; Kong et al., 2009, 2012), with differences in running kinematics and kinetics that have been shown to affect the capacity to discriminate the actual running speed (Nelson et al., 1972; Nigg et al., 1995; Wank et al., 1998; Riley et al., 2008). Contrary to previous studies performed with running individuals (Caramenti et al., 2018, 2019), here we did not find a speed-dependent modulation of the visual-locomotor gain. This could be due to the high PA levels of the participants, which were estimated with the IPAQ questionnaire (MET-min/week: 3956 ± 1777). In fact, PA seems to modulate visual speed perception with individuals running in treadmill-mediated VEs (Caramenti et al., 2019). In particular, previous studies have shown that when used to move within a range of velocities, people might associate sensory signals related to self-motion (biomechanical and vestibular information, optical flow) to their actual locomotion speed more adequately (Bredin et al., 2005). This could mean that regular exposure to visual flow at certain speeds might help to better calibrate the mapping between motion-evoked visual information and the corresponding kinesthetic and motor information.

Another factor that might have contributed to the underestimation of visual speed is the lack of stereoscopic information. Specifically, some studies have shown that stereopsis can provide information about relative distance not only in the peripersonal space but also at greater observation distances (Palmisano et al., 2010), and that perceived depth tends to be more “compressed” under monocular viewing conditions (Allison et al., 2009). Moreover, stereoscopic cues seem to enhance the precision of perceived speed, thereby helping to estimate the rate of self-motion (Brooks and Rafat, 2015) and the perceived speed of vection (Palmisano et al., 2019).

## Conclusion

Treadmill-mediated VEs may soon become a useful and broadly used tool to enhance people’s engagement and adherence to PA programs. Our results indicate that the design of such environments should definitely take into account the size of the FoV in order to reduce the discrepancy between visual and kinesthetic/efferent information during treadmill locomotion, so as to provide the most natural and engaging feedback possible.

## Data Availability Statement

All datasets generated for this study are included in the manuscript/Supplementary Files.

## Ethics Statement

The studies involving human participants were reviewed and approved by Internal Review Board of the Department of Psychology of the University of Fribourg. The patients/participants provided their written informed consent to participate in this study.

## Author Contributions

MC, PP, J-PB, AD, and CL designed the experiments. MC and AD performed the experiments. MC, AD, and JB performed the statistical analysis. MC wrote the first draft of the manuscript. All authors contributed to the manuscript revision, read, and approved the submitted version.

## Conflict of Interest

PP was employed by Virtual Vehicle Research Center, Graz, Austria. The remaining authors declare that the research was conducted in the absence of any commercial or financial relationships that could be construed as a potential conflict of interest.
