# Supplementary material for: Influence of the Size of the Field of View on Visual Perception While Running in a Treadmill-Mediated Virtual Environment
Source: Front Psychol. 2019 Oct 16;10:2344. doi: 10.3389/fpsyg.2019.02344 (PMC6812648; doi:10.3389/fpsyg.2019.02344)
Supplement: Supplementary file 1 [file Data_Sheet_1.zip › S1_Caption.pdf]

## Supplementary Material: Data of the study.

Martina Caramenti, Paolo Pretto, Claudio L. Lafortuna, Jean-Pierre Bresciani, Amandine Dubois

**Supplementary Table S1.** Visual speed for each running speed (i.e. treadmill speed) in the different Field of View conditions (0 = Full screen, 1 = Peripheral view, 2 = Central view).
